# Health-related Quality of life in 640 head and neck cancer survivors after radiotherapy using EORTC QLQ-C30 and QLQ-H&N35 questionnaires

**DOI:** 10.1186/1471-2407-11-128

**Published:** 2011-04-12

**Authors:** Stephen Wan Leung, Tsair-Fwu Lee, Chih-Yen Chien, Pei-Ju Chao, Wen-Ling Tsai, Fu-Min Fang

**Affiliations:** 1 Department of Radiation Oncology, Yuan's General Hospital, Kaohsiung City, Taiwan; 2 Department of Radiological Technology, Central Taiwan University of Science and Technology, Taichung City, Taiwan; 3 Medical Physics & Informatics Lab., Department of Electronics Engineering, National Kaohsiung University of Applied Sciences, Kaohsiung, Taiwan; 4 Department of Otolaryngology, Chang Gung Memorial Hospital - Kaohsiung Medical Center, Chang Gung University College of Medicine, Kaohsiung, Taiwan; 5 Radiation Oncology, Chang Gung Memorial Hospital - Kaohsiung Medical Center, Chang Gung University College of Medicine, Kaohsiung, Taiwan; 6 Department of Biotechnical Cosmetology, Cheng Shiu University, Kaohsiung, Taiwan

## Abstract

**Background:**

With the advances in modern radiotherapy (RT), many patients with head and neck cancer (HNC) can be effectively cured, and their health-related quality of life (HR-QoL) has become an important issue. In this study, we evaluated the prognosticators of HR-QoL in a large cohort of HNC patients, with a focus on the result from technological advances in RT.

**Methods:**

A cross-sectional investigation was conducted to assess the HR-QoL of 640 HNC patients with cancer-free survival of more than 2 years. Among them, 371 patients were treated by two-dimensional RT (2DRT), 127 by three-dimensional conformal RT (3DCRT), and 142 by intensity-modulated RT (IMRT). The EORTC QLQ-C30 questionnaire and QLQ-H&N35 module were used. A general linear model multivariate analysis of variance was used to analyze the prognosticators of HR-QoL.

**Results:**

By multivariate analysis, the variables of gender, annual family income, tumor site, AJCC stage, treatment methods, and RT technique were prognosticators for QLQ-C30 results, so were tumor site and RT technique for H&N35. Significant difference (*p *< 0.05) of HR-QoL outcome by different RT techniques was observed at 2 of the 15 scales in QLQ-C30 and 10 of the 13 scales in H&N35. Compared with 2DRT, IMRT had significant better outcome in the scales of global QoL, physical functioning, swallowing, senses (taste/smell), speech, social eating, social contact, teeth, opening mouth, dry mouth, sticky saliva, and feeling ill.

**Conclusions:**

The technological advance of RT substantially improves the head-and-neck related symptoms and broad aspects of HR-QoL for HNC survivors.

## Background

Health-related quality of life (HR-QoL) and its assessment have become increasingly important in health care, especially in the field of chronic diseases. Conventionally, the endpoints of medical care for cancer patients usually focused on the so-called survival rate, local control rate, or complication rate. These endpoints were usually assessed from the physician's points of view. These assessments lacked knowledge and understanding of the patients' mental and emotional well being. HR-QoL generally refers to the patient's perception of the effects of the disease and the impact on the patient's daily functioning, and has two fundamental premises. First, it is a multi-dimensional survey incorporating physical, psychological, social, and emotional functional domains. Second, it is subjective and must be self-reporting, according to the patient's own experiences [1].

Determining how to measure and quantify the subjective experience of HR-QoL has been a challenging issue. There are now a variety of well-validated HR-QoL instruments available for use in the field of oncology. Three types of methods have been categorized. They include the generic type, e.g. the Short Form-36 (SF-36), the cancer specific type, e.g. the Functional Assessment of Cancer Treatment (FACT-G), the European Organization of Research and Treatment of Cancer Quality of Life Core Questionnaire, version 3.0 (EORTC QLQ-C30), and the cancer site-specific type, e.g. the head and neck modules in EORTC (EORTC QLQ-HN35), and FACT (FACT-HN) [2-6].

Perhaps in no other group of cancer patients does HR-QoL present as important a role as in HNC patients. This is because they may have obviously debilitating problems with swallowing, speech, and hearing, as well as the psychological effects of loss of function and change in body image [7]. Radiotherapy is one of the most important treatment modalities for HNC patients, either in a definite way or a combination with surgery and/or chemotherapy (C/T). Over the past decade, the advances of RT techniques for treating HNC have emerged from so-called two-dimensional RT (2DRT) to the three-dimensional conformal RT (3DCRT) and intensity-modulated RT (IMRT). 2DRT has proven effective in the treatment of HNC. However, complications associated with irradiation of sensitive normal structures, such as the salivary glands in the path of the irradiation, are still remarkable and often lifelong. The reliance of 3DCRT and IMRT on computed tomography-guided 3D planning allows better delineation of tumor target and organs at risk with clearer radiological visualization of their spatial relations, thus providing a potentially therapeutic benefit of dose escalation to tumor tissue with reduced toxicity to normal tissues [8].

IMRT represents an advanced form of 3DCRT. It employs inverse planning algorithms and iterative computer-driven optimization to generate treatment fields with varying beam intensity. Combinations of intensity-modulated fields produce custom-tailored conformal dose distributions around the tumor, with steep dose gradients at the transition to adjacent normal tissues. Growing reports have shown that the technical and dosimetric superiority of 3DCRT and IMRT over 2DRT can translate into clinical benefits, such as reduced normal tissue toxicity (e.g., parotid gland sparing), improved local control, or even patient survival [9-14].

Radical RT for treating HNC was routinely delivered by 2DRT in our hospital before the introduction of 3DCRT in April 1996. From April 1996 to March 2002, 3DCRT was gradually used to replace 2DRT. After becoming familiar with the techniques of 3DCRT and implementation of the IMRT system by March 2002, the physicians and physicists in our institute began to use the two techniques in treating HNC. In a previous publication, we have reported that HNC survivors had significantly poorer HR-QoL outcomes compared with Taiwanese norms [15,16]. In this study, we further compared the HR-QoL results assessed by the EORTC QLQ-C30 and QLQ-H&N35 modules for HNC survivors who, as a result of technological advances in RT at our institute, were treated with 2DRT, 3DCRT, or IMRT in different time periods.

## Methods

### Study population

This study is a cross sectional investigation, analyzing HR-QoL data of HNC patients who were cancer free when their HR-QoL was assessed during the period from January 2005 to December 2008. Eligibility criteria of patients included 1) pathologically proven HNC at nasopharynx, oral cavity, oropharynx, hypopharynx, or larynx, 2) receiving RT and regular follow-up at the department of radiation oncology at Chang Gung Memorial Hospital - Kaohsiung Medical Center, 3) cancer free survival more than two years after RT, and 4) completion of the self-reported questionnaire. Six hundred and forty HNC patients, treated with definite or postoperative RT, were collected and informed consent was obtained from all of them. They included 371 patients treated by 2DRT and 269 patients by conformal RT (3DCRT: 127 patients, IMRT: 142 patients). Concerning the existence of selection bias, we compared the distributions of sociodemographic characteristics (including age, gender, marital status, and education level) between HNC survivors in the study and all other surviving HNC patients (n = 221) found in the cancer registry database in the department. No statistically significant differences were found between them (data not shown).

Patient characteristics including sociodemographic variables and cancer- or treatment- related variables are listed in Table 1. The cancer stages were according to the staging system of the American Joint Committee on Cancer (AJCC 6^th ^edition) published in 2002. The comorbidity status was recorded according to the Charlson comorbidity index by review of chart and on the basis of self-report [17]. A summary of the primary cancer site included 316 cases (49%) of nasopharyx, 129 (20%) of oral cavity, 75 (12%) of oropharynx, 75 (12%) of hypopharynx, and 45 (7%) of larynx. As regards the sociodemographic information, 86% of them were educated ≦12 years, 53% with an annual family income ≦1.2 million NTD (1USD = 33NTD), 65% not employed, and 19% without a spouse (unmarried or divorced). Four hundred and thirty patients (67%) had AJCC stage III or IV disease and 267 (42%) patients had at least one kind of comorbidity. The major treatment was surgery in 249 (39%) patients and RT alone or plus C/T in 391 (61%) patients. Uneven distribution existed between the 2DRT and 3DCRT/IMRT group. The 3DCRT/IMRT group had a higher distribution in patients with lower annual family income, non-nasopharyngeal cancer site, stage III-IV, surgery, chemotherapy, or shorter survival years. The median (range) follow-up years of patients after treatment when their HR-QoL data were collected were 5.2 (2.8-14.1), 3.9 (2.1-10.3), and 3.1 (2.0-6.5) years in the 2DRT, 3DCRT, and IMRT group, respectively. This study was approved by the appropriate institutional review boards (IRB) of the hospital.

**Table 1:** Patient characteristics (n = 640)

Variables	Total	2DRT	3DCRT	IMRT
Patient number	640	371	127	142
Age, median (range) years	52 (15-87)	52 (15-87)	53 (31-83)	51 (23-79)
Male/female	537/103	297/74	117/10	123/19
Education years				
≦6	245	147	52	46
6~12	305	168	64	73
>12	90	56	11	23
Annual family income, (104 NTD)				
<60	119	53	30	36
60~120	218	125	47	46
≥ 120	303	193	50	60
Marital status				
With spouse	518	298	102	118
Without spouse	122	73	25	24
Employment				
Yes	225	139	36	50
No	415	232	91	92
Cancer sites				
Nasopharynx	316	226	31	59
Oral cavity	129	64	33	32
Oropharynx	75	41	21	13
Hypoppharynx	75	20	28	27
Larynx	45	20	14	11
AJCC stage				
I-II	210	145	26	39
III-IV	430	226	101	103
Radiation dose				
≦70.2Gy	281	126	81	74
>70.2Gy	359	245	46	68
Surgery				
No	391	264	53	74
Yes	249	107	74	68
Chemotherapy				
No	343	237	57	49
Yes	297	134	70	93
Comorbidity score				
0	373	219	66	88
≧1	267	152	61	54
Follow-up years, median	4.3	5.2	3.9	3.1

Abbreviations: RT = radiotherapy; 2DRT = two dimensional RT; 3DCRT = three dimensional conformal RT; IMRT = intensity-modulated RT; 1USD = 33NTD; AJCC = American Joint Committee on Cancer, 6th edition; Comorbidity score was based on Charlson comorbidity index.

### Techniques of RT

#### 2DRT

The detailed portal arrangement and dosing of conventional 2DRT in HNC have been described previously [18,19]. Briefly, 2DRT was given in two phases, namely before and after 44~46.8 Gy of the spinal cord tolerance dose. In the first phase, patients were irradiated by a 6-MV photon beam with a daily fraction of 1.8 or 2.0 Gy (5~6 fractions per week) via bilateral opposing faciocervical fields and one lower anterior cervical field. For definite RT, the target covers the primary tumor with surrounding anatomic area and regional neck lymphatics. For postoperative cases, the surgical tumor and nodal bed as well as the prophylactic risky nodal area were included. In the second phase, the gross tumor was boosted to 64.8~81 Gy in definite RT and tumor bed to 57.6~64.8 Gy in postoperative cases via bilateral opposing photon beams to shield the spinal cord. Residual neck lymph nodes or risky nodal bed area were simultaneously boosted by a 9- or 12-Mev electron beam to 56~79 Gy, depending on the nodal situation.

#### 3DCRT

The immobilization, treatment targets, and dose/fractionation prescription of 3DCRT in treating HNC in our institute primarily followed the guidelines for 2DRT. The Cadplan (Varian, Milpitas, CA) or Pinnacle 3D treatment planning system (Pinnacle3, Philips, Fitchburg, WI) was used. The technical details of 3DCRT in HNC have been addressed [19,20]. For each patient, 5 or 7 coplanar portals were usually designed. Shrinkage of the clinical target volume (CTV) volume was usually performed after the tumoricidal dose of 45.0-50.4 Gy was reached for the microscopic lesions. The 90-95% isodose volume to cover the planning target volume (PTV) with the spinal cord strictly limited below the 60% isodose line was applied.

#### IMRT

The immobilization, target definition and delineation, and dose/fractionation prescription of IMRT for HNSCC treated by combined modality were approximately the same as described above for 3DCRT. We used the Cadplan or Pinnacle treatment planning system to perform the inverse planning and dose optimisation. For each patient, IMRT plans with five or seven coplanar portals were created. The delivery of the plans was performed in Varian machines equipped with dynamic multi-leaf collimators. The dose/fractionation prescription of IMRT primarily followed the guidelines for 3DRT [16,21].

### Instruments of HR-Qo*L*

The Taiwan Chinese versions of the EORTC QLQ-C30 and QLQ-H&N35 questionnaires were obtained from the Quality of Life Unit, EORTC Data Center in Brussels, Belgium [4,6,22]. The EORTC questionnaires were chosen for this research because it is one of the most widely implemented questionnaires, with over 10 years of research invested to develop an integrated, modular approach, as well as utilization of the instrument in international clinical trials, and the Taiwan Chinese version is available and easily completed by our patients [23]. The EORTC QLQ-C30 incorporates a range of HR-QoL issues relevant to a broad range of cancer patients. It has been translated into many languages and validated for many types of cancer, including head-and-neck cancer. It contains five functional scales (physical, role, cognitive, emotional and social), three symptom scales (fatigue, pain, and nausea/vomiting), a global QoL scale, and six single-items (dyspnea, insomnia, appetite loss, constipation, diarrhea, and financial difficulties). The QLQ-H&N35 is a module used for assessing the HR-QoL for head-and-neck cancer patients. It incorporates seven multiple-item scales that assess the symptoms of pain, swallowing ability, senses (taste/smell), speech, social eating, social contact, and sexuality. Also included are six single-item scales, which survey the presence of symptomatic problems associated with teeth, mouth-opening, dry mouth (xerostomia), sticky saliva, coughing, and feeling ill. All scales pertaining to the EORTC QLQ-C30 and QLQ-H&N35 range from zero to 100. A high score for a functional or global HR-QoL scale represents a relatively high/healthy level of functioning or global quality of life, whereas a high score for a symptom scale represents the presence of a symptom or problem(s).

### Statistic analysis

The mean scores and standard deviations of the HR-QoL scales were calculated according to the EORTC QLQ scoring manual [24]. To analyze the correlations between the factors and the HR-QoL scales, general linear model (GLM) multivariate analysis of variance (MANOVA) was used [25]. The GLM-MANOVA approach is used to test the hypothesis of a significant association between a set of interrelated dependent variables (HR-QoL scales) and independent variables. The independent variables analyzed in the present study consisted of five sociodemographic variables: age (<40 *v*. 40~60 *v*. ≥ 60 years), sex (male *v*. female), years of education years (≤6 *v*. 6~12 *v*. >12 years), marital status (with *v*. without spouse), and annual family income (<0.6 *v*. 0.6~1.2 *v*. ≥1.2 million NTD), and six clinical factors: CCI score (0 *v*. ≥1), tumour site (Oral cavity *v*. Oropharynx *v*. Hypopharynx/Larynx *v*. Nasopharynx), AJCC stage (stage I-II *v*. III-IV), treatment methods (surgery + RT *v*. surgery + RT + C/T *v*. RT ± C/T), RT technique (2DRT *v*. 3DCRT *v*. IMRT), and follow-up years (2~3 *v*. 3~5 *v*. > 5).

GLM-MANOVA was performed for QLQ-C30 and H&N35 separately and in the following two steps. First, to investigate the association of a given factor with HR-QoL scales, a univariate analysis was conducted to establish whether the factor was associated significantly with any of the HR-QoL scales. Wilks' λ was used to test the impact of each variable included in the model. All variables were entered into the multi-factor model. In case of a significant association between a factor and all HR-QoL scales taken together, a second ANOVA was performed to investigate the association between that prognostic factor and each HR-QoL scale separately, with post-hoc testing using the Bonferroni method with a *p*-value <0.05 from the two-sided test regarded to be statistically significant.

Being the most concerned scales of HR-QoL in the study, the global QoL and xerostomia were further analyzed by multiple linear regression models to explore their associated prognosticators, respectively. All the data processing was performed using the statistic software SPSS for Windows (version 15.0; SPSS Inc., Chicago, IL).

## Results

### Outcomes of HR-QoL

The mean score for global QoL was 54.6. The value of the five scales of functioning ranged from 75.1 (social functioning) to 86.4 (role functioning). The highest symptom score on QLQ-C30 was for fatigue, followed by financial problems and insomnia. In the H&N35 module, dry mouth, sticky saliva, and tooth problems ranked as the three worst symptoms. (Table 2)

**Table 2:** Calculated scores of EORTC QLQ-C30 and H&N35 scales for head and neck cancer survivors

Scales	Mean (SD)	Median	Range
QLQ-C30			
Global quality of life	54.6 (19.9)	50.0	0.0-100.0
Physical functioning	84.5 (16.5)	86.7	11.1-100.0
Role functioning	86.4 (21.0)	100.0	0.0-100.0
Emotional functioning	77.9 (20.0)	75.0	0.0-100.0
Cognitive functioning	78.4 (19.8)	83.3	0.0-100.0
Social functioning	75.1 (24.2)	66.7	0.0-100.0
Fatigue	29.2 (20.4)	33.3	0.0-100.0
Nausea/Vomiting	9.3 (17.5)	0.0	0.0-100.0
Pain	21.9 (22.4)	16.7	0.0-100.0
Dyspnea	15.2 (21.6)	0.0	0.0-100.0
Insomnia	26.3 (25.9)	33.3	0.0-100.0
Appetite loss	19.9 (24.6)	0.0	0.0-100.0
Constipation	19.1 (23.7)	33.3	0.0-100.0
Diarrhea	14.0 (19.9)	0.0	0.0-100.0
Financial problems	26.4 (27.1)	33.3	0.0-100.0
H&N35			
Pain	19.1 (20.6)	16.7	0.0-100.0
Swallowing	30.2 (24.4)	25.0	0.0-100.0
Senses (taste/smell)	26.9 (27.7)	25.0	0.0-100.0
Speech	28.0 (25.9)	22.2	0.0-100.0
Social eating	28.5 (26.9)	25.0	0.0-100.0
Social contact	20.4 (23.5)	13.3	0.0-100.0
Sexuality	26.0 (27.7)	33.3	0.0-100.0
Teeth	38.9 (29.8)	33.3	0.0-100.0
Opening mouth	32.8 (31.6)	33.3	0.0-100.0
Dry mouth	48.3 (31.0)	33.3	0.0-100.0
Sticky saliva	40.7 (30.6)	33.3	0.0-100.0
Coughing	29.3 (25.4)	33.3	0.0-100.0
Feeling ill	29.3 (25.3)	33.3	0.0-100.0

Abbreviations: EORTC QLQ-C30: European Organization for Research and Treatment of Cancer Quality of Life Questionnaire C30; H&N35: Head and Neck Module; SD: standard deviation.

### Variables associated with HR-QoL outcomes

In the first step of the GLM-MANOVA, the association between the independent variables (five sociodemographic and six clinical variables) and the dependent variables (15 scales of QLQ-C30) was investigated (one-factor model, Table 3). This analysis showed that 10 of the 11 variables (except the length of follow-up) (*p *< 0.05) were associated with the overall outcome on QLQ-C30. The 11 variables were then entered into the multifactor model analysis, which indicated that the variables of gender, annual family income, tumor site, AJCC stage, treatment methods, and RT technique remained significant. The same statistical procedures were repeated for the analysis of the association between the 11 independent variables and the scales of H&N35. In a one-factor model, four of the five sociodemographic variables and four of the six clinical variables (except CCI and the length of follow-up) were significantly (*p *< 0.05) associated with the overall outcome on H&N35. In the multifactor model, only tumor site and RT technique remained significant (Table 3).

**Table 3:** GLM-MANOVA test of the overall effect of the sociodemographic and clinical variables on the EORTC QLQ-C30 and H&N35 scales

	QLQ-C30				QLQ-H&N35			
	One-factor model*		Multifactor model**		One-factor model*		Multifactor model**	

**Variable**	**Wilk's λ**	**p **	**Wilk's λ**	**p**	**Wilk's λ**	**p **	**Wilk's λ**	**p**

Sociodemographic variables								
Age: <40 v 40~60 v ≥ 60 years	0.922	0.016	0.942	NS	0.926	0.013	0.939	NS
Gender: female v male	0.890	<0.01	0.877	<0.01	0.919	0.018	0.963	NS
Education years: ≤ 6 v 6~12 v >12	0.914	0.011	0.928	NS	0.920	0.033	0.941	NS
Marital status: with v without spouse	0.958	0.042	0.951	NS	0.975	NS	0.968	NS
Annual family income: < 0.6 v 0.6~1.2 v ≥ 1.2 (million NTD)	0.845	<0.01	0.896	0.032	0.866	<0.01	0.910	NS
Clinical variables								
CCI score: 0 v ≥ 1	0.937	0.001	0.910	NS	0.965	NS	0.935	NS
Tumor site: Oral cavity v Oropharynx v Hypopharynx/Larynx v Nasopharynx	0.805	<0.01	0.828	<0.01	0.618	<0.01	0.824	<0.01
AJCC stage: stage I-II v III-IV	0.870	<0.01	0.863	<0.01	0.880	<0.01	0.869	NS
Treatment methods: S + RT v S + RT+ C/T v RT±C/T	0.865	<0.01	0.845	<0.01	0.832	<0.01	0.906	NS
RT technique: 2DRT v 3DCRT v IMRT	0.888	<0.01	0.876	<0.01	0.869	<0.01	0.829	<0.01
Follow-up years: 2~3 v 3~5 v > 5	0.937	NS	0.928	NS	0.933	NS	0.938	NS

*: The one factor model: only one independent variable was entered into the model. **: The multifactor model: all mentioned variables were entered as independent variables in the model. Abbreviations: GLM-MANOVA: general linear model multivariate of variance; EORTC QLQ-C30: European Organization for Research and Treatment of Cancer Quality of Life Core questionnaire; H&N35: Head and Neck Module; NTD: New Taiwan Dollar (1 USD = 33 NTD); CCI: Charlson comorbidity index; AJCC: American Joint of Cancer Committee published in 2002; S: surgery; RT: radiotherapy; C/T: chemotherapy; 2DRT: two dimensional RT; 3DCRT: three dimensional conformal RT; IMRT: intensity modulated RT; NS: not significant.

The variables specifically associated with global QoL and xerostomia were further analyzed. As demonstrated by linear regression model in Table 4 we observed a significant trend that survivors with CCI ≥ 1 (*β *= 5.2, p = 0.034), tumor sits at nasopharynx (*β*=-9.1, p = 0.034), or treated by 2DRT (*β*=-12.2, p < 0.01) had a higher probability to report a high level of xerostomia. Meanwhile, survivors with family annual income ≧1.2 million NTD (*β *= 5.2, p < 0.01), CCI = 0 (*β*=-6.1, p < 0.01), or treated by 3DCRTor IMRT (*β *= 7.7, p < 0.01) had a higher probability of reporting a better global QoL.

**Table 4:** Multiple linear regression analysis for global quality of life and xerostomia

	Xerostomia			Global quality of life		
**Variable**	**β**	**SE**	**p**	**β**	**SE**	**p**

Sociodemographic variables						
Age: <40 v 40~60 v ≥60 years	-0.7	2.5	NS	-0.2	1.6	NS
Gender: female v male	-1.7	3.5	NS	0.5	2.2	NS
Education years: ≤ 6 v 6~12 v >12	1.2	3.6	NS	3.2	1.3	NS
Marital status: with v without spouse	-2.6	3.1	NS	-3.0	2.0	NS
Annual family income: <1.2 v ≥1.2 (million NTD)	-4.0	2.5	NS	5.2	1.6	<0.01
Clinical variables						
CCI score: 0 v ≥ 1	5.2	2.5	0.034	-6.1	1.6	<0.01
Tumor site: Nasopharynx v others	-9.1	4.3	0.036	-0.9	2.7	NS
AJCC stage: stage I-II v III-IV	1.5	3.1	NS	0.2	2.0	NS
Treatment methods: S + RT v S + RT+ C/T v RT±C/T	3.0	3.7	NS	-1.8	2.3	NS
RT technique: 2DRT v 3DCRT/IMRT	-12.2	2.7	<0.01	7.7	1.7	<0.01
Follow-up years: 2~3 v 3~5 v > 5	0.2	2.9	NS	1.6	1.9	NS

β: un-standardized regression coefficient; SE: standard error; R2 = 0.072 in the model for xerostomia; R2 = 0.077 in the model for global quality of life; NTD: New Taiwan Dollar (1 USD = 33 NTD); CCI: Charlson comorbidity index; AJCC: American Joint of Cancer Committee published in 2002; S: surgery; RT: radiotherapy; C/T: chemotherapy; 2DRT: two dimensional RT; 3DCRT: three dimensional conformal RT; IMRT: intensity modulated RT; NS: not significant.

### Tumor site and HR-QoL outcome

Significant difference (*p *< 0.05) of HR-QoL outcome at different tumor sites was observed at 11 of the 15 scales in QLQ-C30 and 10 of the 13 scales in H&N35 (Table 5). Compared with nasopharyngeal cancer survivors, three worse scales (social eating, social contact, and opening mouth) were significantly observed on oral cancer survivors, so were six scales (appetite loss, pain, swallowing, speech, social eating, and social contact) on oropharyngeal cancer survivors and eight scales (physical functioning, role functioning, dyspnea, constipation, swallowing, speech, social contact, coughing) on hypopharyneal/laryngeal cancer survivors. Meanwhile, compared with oral cavity survivors, five worse scales (cognitive functioning, fatigue, nausea/vomiting, appetite loss, and pain) were observed in oropharyngeal cancer survivors, and five worse scales (physical functioning, nausea/vomiting, dyspnea, speech, and coughing) in hypopharyneal/laryngeal cancer survivors. On the contrast, better results in opening mouth and dry mouth were observed in hypopharyneal/laryngeal cancer survivors compared with the other survivors.

**Table 5:** The comparisons of EORTC QLQ-C30 and H&N35 scales for head and neck cancer survivors at different tumor sites

	Nasopharynx (n = 316)	Oral cavity (n = 129)	Oropharynx (n = 75)	Hypropharynx &Larynx(n = 120)	
	**Mean (SD)**	**Mean (SD)**	**Mean (SD)**	**Mean (SD)**	**p **

EORTC QLQ-C30					
Global quality of life	53.7 (20.4)	56.1 (16.7)	55.2 (21.6)	54.8 (20.9)	NS
Physical functioning	85.7 (16.3)	86.4 (13.8)e	82.2 (16.9)	80.9 (18.7)c	0.015
Role functioning	88.7 (19.5)	87.0 (19.1)	83.6 (22.1)	81.2 (25.0)c	<0.01
Emotional functioning	78.0 (19.7)	79.9 (18.6)	75.3 (22.7)	77.0 (20.8)	NS
Cognitive functioning	77.8 (18.7)	82.8 (19.2)d	75.1 (23.0)	76.9 (20.9)	0.025
Social functioning	77.0 (23.2)	75.4 (22.8)	73.1 (27.0)	71.0 (25.8)	NS
Fatigue	29.6 (19.8)	24.9 (21.2)d	32.7 (19.2)	30.4 (21.4)	0.037
Nausea/Vomiting	8.4 (15.6)	6.2 (14.2)d, e	13.5 (22.5)	12.4 (20.8)	<0.01
Pain	20.8 (21.5)	19.2 (23.1)	26.8 (21.9)	24.9 (23.8)	0.038
Dyspnea	12.7 (19.9)	12.4 (20.4)e	17.8 (23.5)	23.6 (24.0)c	<0.01
Insomnia	25.1 (26.3)	23.1 (25.5)	30.1 (22.8)	30.7 (26.5)	0.049
Appetite loss	17.0 (23.8)	17.9 (24.3)d	30.7 (26.0)b	23.1 (24.1)	<0.01
Constipation	16.5 (22.4)	18.4 (22.4)	23.7 (26.8)	24.0 (25.3)c	<0.01
Diarrhea	13.4 (18.9)	12.2 (18.6)	16.4 (26.0)	15.9 (19.3)	NS
Financial problems	23.4 (26.4)	26.9 (25.7)	31.9 (29.9)	30.5 (27.9)	0.023
EORTC QLQ-HN35					
Pain	17.0 (18.3)	17.3 (22.9)d	27.4 (20.7)b	21.5 (22.5)	<0.01
Swallowing	26.8 (22.4)	30.6 (25.4)	38.0 (23.6)b	33.8 (27.5)c	<0.01
Senses (taste/smell)	27.2 (27.0)	22.1 (26.8)	26.4 (27.4)	31.3 (30.1)	NS
Speech	23.0 (23.3)	25.6 (21.8)e	32.0 (27.1)b	41.3 (30.7)c	<0.01
Social eating	24.1 (24.9)	33.2 (27.0)a	35.4 (27.8)b	30.7 (29.5)	<0.01
Social contact	15.4 (19.9)	24.0 (23.2)a	23.9 (26.7)b	27.5 (27.4)c	<0.01
Sexuality	23.8 (27.6)	24.3 (24.4)	30.5 (29.4)	31.3 (29.5)	0.035
Teeth	38.1 (28.3)	41.0(31.8)	42.7 (30.2)	36.1 (31.3)	NS
Opening mouth	30.1 (29.7)	43.9 (34.2)a, e	37.8 (32.9)f	25.1 (29.4)	<0.01
Dry mouth	53.5 (31.3)	45.9 (30.7)	49.2 (30.6)f	36.6 (27.5)c	<0.01
Sticky saliva	42.8 (31.7)	37.6 (28.4)	46.3 (29.8)	35.0 (29.8)	0.024
Coughing	25.9 (23.9)	23.9 (23.3)e	31.5 (27.4)f	43.1 (25.4)c	<0.01
Feeling ill	29.6 (25.5)	24.8 (23.7)	31.5 (25.1)	32.1 (26.0)	NS

a : p < 0.05, Oral cavity compared with Nasopharynx; b : p < 0.05, Oropharynx compared with Nasopharynx; c : p < 0.05, Hypropharynx & Larynx compared with Nasopharynx; d : p < 0.05, Oral cavity compared with Oropharynx; e : p < 0.05, Oral cavity compared with Hypropharynx & Larynx; f : p < 0.05, Oropharynx compared with Hypropharynx & Larynx

### RT techniques and HR-QoL outcome

Significant difference (*p *< 0.05) of HR-QoL outcome by different RT techniques was observed at 2 of the 15 scales in QLQ-C30 and 10 of the 13 scales in H&N35 (Table 6). Compared with 2DRT, IMRT had significant better outcome in the scales of global QoL, physical functioning, swallowing, senses (taste/smell), speech, social eating, social contact, teeth, opening mouth, dry mouth, sticky saliva, and feeling ill. Only three scales (teeth, dry mouth, and sticky saliva) with better results were observed in 3DCRT compared with 2DRT. IMRT had better scores in most scales compared with 3DCRT, but without reaching statistically significant difference.

**Table 6:** The comparisons of EORTC QLQ-C30 and H&N35 scales for head and neck cancer survivors by different RT techniques

	2DRT (n = 371)	3DCRT (n = 127)	IMRT (n = 142)	
	**Mean (SD)**	**Mean (SD)**	**Mean (SD)**	**p **

EORTC QLQ-C30				
Global quality of life	51.7 (19.9)	56.2 (18.6)	60.7 (19.7)b,	<0.01
Physical functioning	83.1 (17.7)	86.2 (14.1)	86.7 (14.6)b	0.039
Role functioning	85.6 (22.2)	86.7 (19.4)	88.1 (19.1)	NS
Emotional functioning	76.6 (20.8)	79.8 (18.0)	79.6 (19.8)	NS
Cognitive functioning	77.2 (20.7)	79.9 (16.6)	80.0 (20.1)	NS
Social functioning	73.4 (26.0)	76.7 (20.3)	78.1 (22.0)	NS
Fatigue	31.0 (21.1)	26.9 (16.8)	26.5 (21.0)	NS
Nausea/Vomiting	10.3 (19.2)	7.7 (13.6)	8.2 (15.5)	NS
Pain	23.9 (24.1)	18.5 (16.9)	19.7 (21.7)	NS
Dyspnea	16.2 (23.4)	15.8 (18.8)	12.1 (18.7)	NS
Insomnia	26.9 (27.5)	25.6 (22.1)	25.4 (25.0)	NS
Appetite loss	21.7 (26.7)	18.8 (20.9)	16.4 (21.2)	NS
Constipation	19.8 (25.2)	19.2 (22.5)	17.1 (20.4)	NS
Diarrhea	14.8 (21.3)	12.4 (17.8)	13.3 (17.6)	NS
Financial problems	27.1 (28.1)	23.1 (24.1)	27.6 (26.7)	NS
EORTC QLQ-HN35				
Pain	20.8 (22.6)	18.4 (16.7)	15.2 (17.7)	NS
Swallowing	33.3 (25.6)	28.0 (22.4)	23.9 (21.5)b	<0.01
Senses (taste/smell)	29.4 (29.0)	26.3 (28.1)	20.6 (22.7)b	<0.01
Speech	29.6 (26.4)	29.5 (25.7)	22.4 (24.0)b	0.013
Social eating	31.0 (27.5)	28.5 (27.0)	21.9 (24.3)b	<0.01
Social contact	21.0 (24.1)	22.7 (24.7)	16.6 (20.3)b	<0.01
Sexuality	27.4 (28.9)	27.0 (28.0)	21.5 (23.8)	NS
Teeth	43.2 (30.6)	34.7 (29.1)a	31.2 (26.0)b	<0.01
Opening mouth	36.5 (31.7)	30.2 (30.4)	25.4 (31.1)b	<0.01
Dry mouth	54.0 (31.9)	43.8 (28.2)a	41.0 (27.9)b	<0.01
Sticky saliva	45.1 (32.3)	35.8 (26.1)a	33.6 (27.9)b	<0.01
Coughing	31.0 (26.9)	29.5 (24.2)	24.5 (21.7)	NS
Feeling ill	32.3 (26.3)	26.3 (23.0)	24.0 (23.2)b	<0.01

Abbreviations: RT = radiotherapy; 2DRT = two dimensional RT; 3DCRT = three dimensional conformal RT; IMRT = intensity-modulated RT; a: p < 0.05, 3DCRT compared with 2DRT; b: p < 0.05, IMRT compared with 2DRT

## Discussion

The treatment fields of conventional 2DRT for HNC are usually large and anatomic structures situated nearby the tumors, such as the salivary glands, inner ear, oral cavity, and temporomandibular joints, are exposed to almost the same irradiated dose as the treatment target sites. We have previously used the Taiwan Chinese version of SF-36 to investigate the HR-QoL of oral cancer or nasopharyngeal survivors treated by conventional RT, finding that these HNC survivors scored significantly worse in most of the eight functional domains of SF-36 than Taiwanese norms [15,16]. However, SF-36 is not specific and sensitive enough as a HR-QoL instrument to discriminate the effect differences for head- and neck-related problems between different treatment modalities. In the present study, we used the EORTC QLQ-C30 and H&N35 to assess HR-QoL for HNC patients. The Taiwan Chinese version of the questionnaires has been previously tested in HNC patients by Chie et al. [23]. They found the Cronbach's alpha coefficients of all scales of the two questionnaires were ≧ 0.70, except that for cognitive functioning, and the correlation of scales that measure similar dimensions of the QLQ-C30 and the SF-36 was moderate to high, whereas that of the H&N35 and the QLQ-C30 or the SF-36 was moderate to low.

Growing reports have shown that the dosimetric superiority of 3DCRT/IMRT applied in HNC patients can not only preserve salivary functions, but also improve local control, and even patient survival [10-12]. The current study presented with a large cohort to compare HR-QoL between HNC survivors receiving different RT techniques applied at a single institute during different time periods, adjusting for major sociodemographic and medical variables that affect these measures. Although heterogeneity existed in the comparing groups, the potential bias of patient selection was reduced, and the contributions of the different RT techniques in producing particular HR-QoL outcomes were highlighted. We demonstrated that the use of IMRT significantly improved HR-QoL for HNC survivors as compared with those treated by conventional 2DRT.

Xerostomia related symptoms were usually cited as the most prevalent complications in HNC survivors post RT and patient-reported xerostomia has been found to significantly correlate with mean dose to the parotid glands and the minor salivary glands in the oral cavity [26]. In a matched comparison of 67 pairs of HNC survivors treated by 2DRT versus IMRT, Graff et al. observed the major advantages of IMRT were on oral symptoms, especially salivary dysfunction and oral discomfort [27]. Similarly, a cross sectional survey of 163 HNC survivors by van Rij et al. also revealed that parotid sparing IMRT for HNC patients improved xerostomia related HR-QoL compared to 2DRT both in rest and during meals [28]. With the large cohort of 640 HNC survivors in current study, we confirmed that RT technique is the determining variable affecting xerostomia in HNC survivors and affirmed that modern RT, especially for IMRT, not only reduced the oral related symptoms but also the global QoL.

However, the direct cause-effect relationship between xerostomia and broad aspect of HR-QoL in HNC patients after RT has not been established yet. The improvement of IMRT in HR-QoL might not be only through reducing xerostomia by salivary glands sparing, but also reducing the volume of other non-target organs receiving a high dose. Some reports have shown that radiation-induced dysphagia also plays an important role in HR-QoL domains [28-30]. Dysphagia is usually multifactorial and strongly associated with xerostomia. It has become increasingly important to identify the anatomical structures that are involved in swallowing problems after RT. A cross-sectional study of 81 patients with oropharyngeal cancer, reported by Levendag et al., showed that the probability of swallowing complaints is significantly associated with the mean total radiation dose in the superior and middle pharyngeal constrictor muscle [29]. Our data confirmed this hypothesis that the use of 3DCRT/IMRT also reduced other head-and-neck-related symptom scales to some extent. The inter- and intra-scale correlations of EORTC QLQ-C30 and QLQ-H&N35 were significantly high in our series [data not shown], which means the symptomatic problems improved by 3DCRT/IMRT are inter-correlative and might converge and reflect to the expression of patients' global QoL.

The comparisons between 3DCRT and IMRT were mainly on the dosimetric distribution in the literature, and the information concerning the clinical advantage attributed to the evolution from 3DCRT to IMRT in HNC patients is still limited and controversial. In our previous study exclusively focusing on nasopharyngeal cancer, a significant reduction of 25-30% of the mean dose to the normal structures such as parotid glands and oral cavity was created by IMRT compared with 3DCRT. However, comparing their longitudinal changes of HR-QoL, we did not find a significant difference at most time points except 3 months after RT [31]. Current study echoes this finding that the positive advantage of IMRT over 3DCRT in QoL is ambiguous and marginal in HNC survivors. In contrast, a non-randomized prospective comparison between HNC patients treated by 3DCRT or IMRT demonstrated that IMRT resulted in a significant reduction of patient- and observer-rated xerostomia, as well as other head and neck symptoms, compared with standard 3DCRT [32]. With locally advanced disease treated by aggressive combined modality in most of our subjects, we found that the dosimetric improvement for 3DCRT compared with IMRT might not be sufficiently large to demonstrate any significant difference in HR-QoL.

Besides radiation technique, socioeconomic status, comorbidity, and tumor site were also found to be significant prognosticators on HR-QoL outcome. With the relatively heterogeneous nature of HNC patients and experienced treatments including varying tumor stages, sites, and the frequently diverse treatment modalities applied and involved administering institutions, the factors affecting the HR-QoL after treatment for HNC patients usually appear to be somewhat discordant and complicated in the literature. For example, Hammerlid et al. found those HNC survivors with tumor located at larynx, aged below 65 years, or female patients had significantly better HR-QoL than their counterparts three years after treatment [33] and de Graeff et al. reported female sex, higher cancer stage, and combination treatment were found to be associated with more symptomatic problems and worse HR-QoL [34].

Although being comprehensive and well validated with recognized internal consistency and reliability, EORTC QLQ-C30 and QLQ-H&N35 still have some limitations in the interpretation of HR-QoL of HNC patients, because they do not deal with some specific but common late sequelae, such as deafness, otitis media, symptoms from temporal lobe necrosis, or radiation neuropathy, or hypopituitarism in nasopharyngeal cancer survivors. A tumor site-specific assessment tool of HR-QoL might provide more specific and sensitive information to discriminate site-related differences of HR-QoL in HNC patients with various tumor sites treated by different strategies. Furthermore, without pre-treatment HR-QoL data available in our cohort, potential selection bias might still exist in the cross sectional study, though the confounding variables adjusted by multivariate analysis. A longitudinal study assessing the changes of HR-QoL will be justified to more accurately detect the differences between the groups.

## Conclusions

With the advance of modern RT technology, head-and-neck related symptoms after RT could be significantly reduced and reflected to the improvement of broad aspects of HR-QoL in HNC survivors. However, there may still be some undetected factors, which are related to global QoL or some specific functional domains, to be explored in future investigation.

## Abbreviations

HR-QoL: health-related quality of life; HNC: head and neck cancer; EORTC QLQ-C30: European Organization for Research and Treatment of Cancer Quality of Life Core questionnaire; H&N35: Head and Neck Module; GLM-MANOVA: general linear model multivariate of variance; NTD: New Taiwan Dollar (1 USD = 33 NTD); CCI: Charlson comorbidity index; AJCC: American Joint of Cancer Committee published in 2002; S: surgery; RT: radiotherapy; C/T: chemotherapy; 2DRT: two dimensional RT; 3DCRT: three dimensional conformal RT; IMRT: intensity modulated RT; β: un-standardized regression coefficient; SE: standard error; NS: not significant.

## Competing interests

The authors declare that they have no competing interests.

## Authors' contributions

SW Leung and TFL: writing of manuscript and study coordinator. FMF: original idea, concept and final revision of manuscript. CC, TFL, WLT and PJC: design and development of study. WLT: statistical analysis. All authors read and approved the final manuscript

## Pre-publication history

The pre-publication history for this paper can be accessed here:

http://www.biomedcentral.com/1471-2407/11/128/prepub

## References

[B1] List M Stracks J Evaluation of quality of life in patients definitively treated for squamous carcinoma of the head and neck Curr Opin Oncol 2000 12 215 10.1097/00001622-200005000-00006 10841193

[B2] Ware J Kosinski M Keller S SF-36 physical and mental health summary scales: a user's manual 1994

[B3] Ware J Jr Sherbourne C The MOS 36-item short-form health survey (SF-36): I. Conceptual framework and item selection Med Care 1992 30 473 483 10.1097/00005650-199206000-00002 1593914

[B4] Aaronson N Ahmedzai S Bergman B The European Organization for Research and Treatment of Cancer QLQ-C30: a quality-of-life instrument for use in international clinical trials in oncology J Natl Cancer Inst 1993 85 365 10.1093/jnci/85.5.365 8433390

[B5] Cella D Tulsky D Gray G The Functional Assessment of Cancer Therapy scale: development and validation of the general measure J Clin Oncol 1993 11 570 8445433 10.1200/JCO.1993.11.3.570

[B6] Bjordal K Hammerlid E Ahlner-Elmqvist M Quality of life in head and neck cancer patients: validation of the European Organization for Research and Treatment of Cancer Quality of Life Questionnaire-H&N35 J Clin Oncol 1999 17 1008 10071296 10.1200/JCO.1999.17.3.1008

[B7] Amdur R Parsons J Mendenhall W Postoperative irradiation for squamous cell carcinoma of the head and neck: an analysis of treatment results and complications Int J Radiat Oncol Biol Phys 1989 16 25 36 10.1016/0360-3016(89)90006-0 2912947

[B8] Teo P Ma B Chan A Radiotherapy for nasopharyngeal carcinoma--transition from two-dimensional to three-dimensional methods Radiother Oncol 2004 73 163 172 10.1016/j.radonc.2004.06.005 15542163

[B9] Chao K Wippold F Determination and delineation of nodal target volumes for head-and-neck cancer based on patterns of failure in patients receiving definitive and postoperative IMRT* 1 Int J Radiat Oncol Biol Phys 2002 53 1174 1184 10.1016/S0360-3016(02)02881-X 12128118

[B10] Saarilahti K Kouri M Collan J Intensity modulated radiotherapy for head and neck cancer: evidence for preserved salivary gland function Radiother Oncol 2005 74 251 258 10.1016/j.radonc.2004.11.004 15763305

[B11] Kwong D Pow E Sham J Intensity-modulated radiotherapy for early-stage nasopharyngeal carcinoma Cancer 2004 101 1584 1593 10.1002/cncr.20552 15378492

[B12] Sultanem K Shu H Xia P Three-dimensional intensity-modulated radiotherapy in the treatment of nasopharyngeal carcinoma: the University of California-San Francisco experience Int J Radiat Oncol Biol Phys 2000 48 711 722 10.1016/S0360-3016(00)00702-1 11020568

[B13] Lee N Xia P Quivey J Intensity-modulated radiotherapy in the treatment of nasopharyngeal carcinoma: an update of the UCSF experience Int J Radiat Oncol Biol Phys 2002 53 12 22 10.1016/S0360-3016(02)02859-6 12007936

[B14] Wolden S Chen W Pfister D Intensity-modulated radiation therapy (IMRT) for nasopharynx cancer: update of the Memorial Sloan-Kettering experience Int J Radiat Oncol Biol Phys 2006 64 57 62 10.1016/j.ijrobp.2005.03.057 15936155

[B15] Fang F Tsai W Chien C Chiu H Wang C Health-related quality of life outcome for oral cancer survivors after surgery and postoperative radiotherapy Jpn J Clin Oncol 2004 34 641 10.1093/jjco/hyh118 15613552

[B16] Fang F Chiu H Kuo W Health-related quality of life for nasopharyngeal carcinoma patients with cancer-free survival after treatment Int J Radiat Oncol Biol Phys 2002 53 959 968 10.1016/S0360-3016(02)02838-9 12095563

[B17] Charlson M Pompei P Ales K Mackenzie C A new method of classifying prognostic comorbidity in longitudinal studies: Development and validation J Chro Diseas 1987 40 373 383 10.1016/0021-9681(87)90171-8 3558716

[B18] Fang F Leung S Huang C Combined-modality therapy for squamous carcinoma of the buccal mucosa: treatment results and prognostic factors Head Neck 1997 19 506 512 10.1002/(SICI)1097-0347(199709)19:6<506::AID-HED8>3.0.CO;2-2 9278759

[B19] Fang F Leung S Wang C Computed tomography findings of bony regeneration after radiotherapy for nasopharyngeal carcinoma with skull base destruction: implications for local control Int J Radiat Oncol Biol Phys 1999 44 305 309 10.1016/S0360-3016(99)00004-8 10760423

[B20] Hsiung C Yorke E Chui C Intensity-modulated radiotherapy versus conventional three-dimensional conformal radiotherapy for boost or salvage treatment of nasopharyngeal carcinoma Int J Radiat Oncol Biol Phys 2002 53 638 647 10.1016/S0360-3016(02)02760-8 12062607

[B21] Hsiung C Hunt M Yorke E Intensity-modulated radiotherapy as the boost or salvage treatment of nasopharyngeal carcinoma: The appropriate parameters in the inverse planning and the effect of patient's anatomic factors on the planning results Radiother Oncol 2005 77 53 57 10.1016/j.radonc.2005.04.017 16246743

[B22] Bjordal K Ahlner-Elmqvist M Hammerlid E A prospective study of quality of life in head and neck cancer patients. Part II: Longitudinal data Laryngoscope 2001 111 1440 1452 10.1097/00005537-200108000-00022 11568582

[B23] Chie W Hong R Lai C Ting L Hsu M Quality of life in patients of nasopharyngeal carcinoma: validation of the Taiwan Chinese version of the EORTC QLQ-C30 and the EORTC QLQ-H&N35 Qual Life Res 2003 12 93 98 10.1023/A:1022070220328 12625522

[B24] Fayers P Bottomley A Quality of life research within the EORTC--the EORTC QLQ-C30 Eur J Cancer 2002 38 125 133 10.1016/S0959-8049(01)00448-8 11858978

[B25] Middel B Stewart R Bouma J Sonderen E Heuvel W How to validate clinically important change in health related functional status. Is the magnitude of the effect size consistently related to magnitude of change as indicated by a global question rating? J Eval Clin Pract 2001 7 399 410 10.1046/j.1365-2753.2001.00298.x 11737531

[B26] Eisbruch A Kim H Terrell J Xerostomia and its predictors following parotid-sparing irradiation of head-and-neck cancer Int J Radiat Oncol Biol Phys 2001 50 695 704 10.1016/S0360-3016(01)01512-7 11395238

[B27] Graff P Lapeyre M Desandes E Impact of intensity-modulated radiotherapy on health-related quality of life for head and neck cancer patients: matched-pair comparison with conventional radiotherapy Int J Radiat Oncol Biol Phys 2007 67 1309 1317 10.1016/j.ijrobp.2006.11.012 17289292

[B28] van Rij C Oughlane-Heemsbergen W Ackerstaff A Parotid gland sparing IMRT for head and neck cancer improves xerostomia related quality of life Radiat Oncol 2008 3 41 10.1186/1748-717X-3-41 19068126 PMC2631487

[B29] Levendag P Teguh D Voet P Van der E Dysphagia disorders in patients with cancer of the oropharynx are significantly affected by the radiation therapy dose to the superior and middle constrictor muscle: a dose-effect relationship Radiother Oncol 2007 85 64 73 10.1016/j.radonc.2007.07.009 17714815

[B30] Teguh D Levendag P Noever I Treatment techniques and site considerations regarding dysphagia-related quality of life in cancer of the oropharynx and nasopharynx Int J Radiat Oncol Biol Phys 2008 72 1119 1127 10.1016/j.ijrobp.2008.02.061 18472364

[B31] Fang F Chien C Tsai W Quality of life and survival outcome for patients with nasopharyngeal carcinoma receiving three-dimensional conformal radiotherapy vs. intensity-modulated radiotherapy - a longitudinal study Int J Radiat Oncol Biol Phys 2008 72 356 364 10.1016/j.ijrobp.2007.12.054 18355980

[B32] Vergeer M Doornaert P Rietveld D Intensity-modulated radiotherapy reduces radiation-induced morbidity and improves health-related quality of life: results of a nonrandomized prospective study using a standardized follow-up program Int J Radiat Oncol Biol Phys 2009 74 1 8 10.1016/j.ijrobp.2008.07.059 19111400

[B33] Hammerlid E Taft C Health-related quality of life in long-term head and neck cancer survivors: a comparison with general population norms Brit J cancer 2001 84 149 10.1054/bjoc.2000.1576 11161369 PMC2363699

[B34] Alexander de Graeff J de Leeuw W Gert-Jan Hordijk G Jacques A Long-term quality of life of patients with head and neck cancer Laryngoscope 2000 110 98 106 10.1097/00005537-200001000-00018 10646723

